# Virulence and genome analysis of three historical *Francisella tularensis* ssp. *holarctica* isolates for development of a Type B test panel

**DOI:** 10.3389/fcimb.2025.1679606

**Published:** 2025-12-03

**Authors:** Kevin D. Mlynek, Joshua B. Richardson, Elsie E. Martinez, Ronald G. Toothman, Ju Qiu, Curtis R. Cline, Joel A. Bozue

**Affiliations:** 1 Bacteriology Division, U.S. Army Medical Research Institute of Infectious Diseases (USAMRIID), Frederick, MD, United States; 2 Center for Genome Sciences, U.S. Army Medical Research Institute of Infectious Diseases (USAMRIID), Frederick, MD, United States; 3 Regulated Research Administration Division, U.S. Army Medical Research Institute of Infectious Diseases (USAMRIID), Frederick, MD, United States; 4 Pathology Division, U.S. Army Medical Research Institute of Infectious Diseases (USAMRIID), Frederick, MD, United States

**Keywords:** *Francisella tularensis*, Type B, *holarctica*, rat, animal modeling, tularemia, pathogenicity

## Abstract

*Francisella tularensis* ssp*. tularensis* (Type A) and ssp. *holarctica* (Type B) are the etiological agent of tularemia. Most studies on *F. tularensis* pathogenesis and vaccine development have focused on the Type A Schu S4 strain. However, Type B isolates remain less understood, emphasizing the need for further research. To address this concern and provide well characterized Type B isolates to test future vaccine efficacy, we selected three Type B isolates available in the USAMRIID repository (VT68, strain 425, and strain 503). These strains were chosen based on the original isolation source, the availability of historical *in vivo* data, and genomic sequence data. Strains were characterized for extracellular and intracellular growth, lipopolysaccharide profile via western blot analysis, and LD_50_ values were determined by both murine intranasal challenge and aerosolization of Fischer 344 rats. Strain 425 displayed several attenuation indicators and was completely attenuated in a rat aerosol challenge model. In contrast, VT68 and 503 remained highly virulent in rodent models but displayed some differences in lesion severity. Although *F. tularensis* genomes are known to be highly conserved, genomic analysis revealed multiple inversions and 68 unique genetic differences among these three Type B isolates. From this study, we were able to provide well-characterized Type B *F. tularensis* strains to test future vaccines and therapeutics.

## Introduction

*Francisella tularensis*, the etiological agent of tularemia, is ubiquitously found across the Northern Hemisphere and can cause fatal disease in mammals, including humans. Infection can occur via multiple routes, including arthropod bites, direct contact with contaminated environments or animals, consumption of infected animals or water, and aerosol exposure. The disease manifestation typically coincides with the route of exposure, as glandular and ulceroglandular tularemia are associated with an arthropod bite or direct handling of infected material, while oropharyngeal tularemia occurs from the ingestion of contaminated food or water (Jellison, 1974; Ellis et al., 2002; Sjostedt, 2007). The most severe forms of the disease are typhoidal and pneumonic tularemia, the latter of which can occur from inhalation of *F. tularensis* and is the most likely exposure route if this bacterium is intentionally released (Kaufmann et al., 1997; Dennis et al., 2001; World Health, 2005). Because of its low infectious dose, high pathogenicity, and the ability to aerosolize this organism, *F. tularensis* is considered a Tier 1 select agent by the United States Department of Health and Human Services (Saslaw et al., 1961; Dennis et al., 2001).

*F. tularensis* has been categorized into subspecies (ssp. *tularensis*, ssp. *holarctica*, and ssp*. mediasiatica*) which differ in distribution, ecology, and virulence (Sjostedt, 2005; Svensson et al., 2009; Vogler et al., 2009). Tularemia in humans is caused by the subspecies *F. tularensis* ssp*. tularensis* and ssp. *holarctica*, commonly referred to as Type A and Type B, respectively, and will be used here. Type A strains are regarded as more virulent than Type B strains; however, differences in virulence between strains are becoming more apparent (Dennis et al., 2001; Kugeler et al., 2009; Johansson and Petersen, 2010; Bachert et al., 2021). Type A isolates are endemic to North America and are often associated with tick vectors in arid environments (Jellison, 1974; Staples et al., 2006). Type B isolates are distributed throughout much of the Northern Hemisphere, including North America and parts of both Europe and Asia, with mosquitos being considered the main factor in localized outbreaks (Abdellahoum et al., 2020). However, ticks have also been shown to carry Type B strains and lead to disease in humans (CDC, 1984; Petersen et al., 2009).

Although arthropods are largely involved with the transmission of *F. tularensis*, it is still an understudied topic. Initially, it was believed that mosquitoes were only capable of transmitting the disease and carrying the bacteria transiently (Petersen et al., 2009). However, evidence now indicates that mosquitoes may transstadially transmit *F. tularensis* from larvae to adult form (Lundstrom et al., 2011). More recently, mice were shown to develop tularemia when injected with adult mosquito homogenate that were exposed to *F. tularensis* as larvae. This study suggests that mosquitoes could maintain *F. tularensis* from an aquatic environment and transmit it to a susceptible host (Backman et al., 2015). In comparison, it believed that ticks could maintain *F. tularensis* for long periods of time through all stages of their life cycle (larvae, nymph, and adult) (Hopla, 1974; Coburn et al., 2015; Mani et al., 2015).

The least prevalent subspecies, *F. tularensis* ssp*. mediasiatica*, has only been detected in parts of Central Asia (Kazakhstan and Turkmenistan) and the Altai region of Russia (Keim et al., 2007; Timofeev et al., 2017). To date, there have been no reports of human tularemia cases caused by this subspecies, although *F. mediasiatica* exhibits high virulence in mice and guinea pigs (Timofeev et al., 2020). The inclusion of *F. novicida* as a subspecies of *F. tularensis* is controversial and debated in the literature (Johansson et al., 2010; Kingry and Petersen, 2014). Historically, *F. novicida* has been endemic to the United States, but more recently, isolates have been identified clinically in Asia and Australia (Keim et al., 2007; Tuanyok et al., 2012). F*. novicida* is typically considered avirulent in humans and is routinely used Biosafety Level (BSL)-2 laboratories as a surrogate for more virulent isolates. When infections with *F. novicida* have occurred in humans it was typically seen in immunosuppressed individuals (Gavina et al., 2023). However, occasional human infections have been reported in otherwise healthy people but usually involved near drowning or exposure to contaminated water (Brett et al., 2012; Whitehouse et al., 2012; Kingry and Petersen, 2014).

Currently, no approved tularemia vaccine is available in the United States or European Union. Therefore, current efforts in the public health and biodefense communities are being invested to address this gap. A successful tularemia vaccine would ideally protect against a wide variety of *F. tularensis* strains, including both Type A and B subspecies. We previously devised a test panel targeted mainly at diverse Type A isolates (Bachert et al., 2021), but there is a need to expand this panel to include Type B isolates that are genetically distinct from one another. To begin to address this concern and provide well-characterized Type B isolates to test vaccine efficacy, we selected three strains for this study based on the original isolate source, availability of historical *in vivo* data, and genomic sequence data. Strain 425 was isolated from a *Dermacentor andersoni* tick in the United States (Bell et al., 1955) and has been previously characterized for virulence in mice and non-human primates (NHPs) by aerogenic exposure (Schricker et al., 1972; Fritz et al., 2014). Similarly, strain 503 was isolated from an Ixodid tick (*Dermacentor pictus* Herm) but is of Eurasian origin (southern Moscow, Russia) (Olsufiev et al., 1959) and has been used as a challenge strain to test potential vaccines in multiple countries (Emel’Ianova, 1957; Tigertt, 1962; Timofeev et al., 2020). Furthermore, both of these Type B strains were serially passaged in guinea pigs after the initial isolate to select for virulence traits by source countries (Bell et al., 1955; Olsufiev et al., 1959). Finally, VT68 was included in this study given the unusual environmental source (muskrat) traced back to an outbreak in 1968 in the United States (Young et al., 1969).

In the current study, we expanded upon these characterized Type B isolates of *F. tularensis* to facilitate testing of new medical countermeasures against pneumonic tularemia. The 425 strain obtained from the USAMRIID repository was found to exhibit a minor growth defect when cultured in Chamberlain’s Defined Medium (CDM) compared to other strains, displayed mild attenuation in a pneumonic murine model, and, unexpectedly, was completely attenuated by aerosol exposure in a rat model. In contrast, the VT68 and 503 strains remained virulent in both rodent models, although differences in histopathological severity were found when comparing the strains in the rat aerosol model of infection. Genomic analysis revealed that each of these isolates was reasonably diverse from one another and representative of a different subclade of Type B isolates, which prompted us to perform an in-depth analysis. Overall, this study provides a framework and dataset to allow informed medical countermeasure testing of Type B strains to thoroughly evaluate future product efficacy for pneumonic tularemia against multiple strains from the *F. tularensis* subspecies.

## Materials and methods

### Bacterial strains and culture conditions

*F. tularensis* ssp*. tularensis* and ssp. *holarctica* strains used in this study are listed in **Table 1** and were obtained from the Biodefense Reference Material Repository (BRMR) housed at USAMRIID. *F. tularensis* was cultured on enriched chocolate agar plates (Remel) at 37°C. For liquid culture, either brain heart infusion broth (BHI) supplemented with IsoVitaleX (Becton-Dickinson, Cockeysville, MD) or CDM (Chamberlain, 1965) at pH 6.2 were used. The pH of BHI supplemented IsoVitaleX was measured and was found to range from ~6.3 to 7.0. At the study onset, it was confirmed that equal CFU (colony forming unit) values by plating were obtained for each isolate when normalized to a known OD_600_ (data not shown). No statistical differences were observed (p = 0.41).

**Table 1:** Strains used in this study.

Strain	BRMR ID	Description	GenBank	Reference
Schu S4	FRAN244	Isolated from a human ulcer, Ohio, USA (1941) BEI (NR-10492)	CP073128	Eigelsbach, 1951
2015321842	FRAN255	Isolated from human (male) pleura, Kentucky, USA (2015)	CP073125	Bachert, 2021 (Bachert et al., 2021)
VT68	FRAN025	Isolated from muskrat spleen during outbreak in Vermont, USA (1968)	CP010288	Bell, 1955, JID (Bell et al., 1955)
425	FRAN029	Isolated from *Dermacentor andersoni* in Montana, USA (1941)	CP010289	Young et al., 1969, NEJM (Young et al., 1969)
503	FRAN045	Isolated from *Dermacentor pictis* in Moscow, USSR (1949)	NZ_LVKX01000000	Olsuf’ev, 1959. J. Hyg. Epidemiol. Microbiol. Immunol (Olsufiev et al., 1959).

### Generation of growth curves

*F. tularensis* strains were resuspended in PBS (~5 mL; phosphate buffered saline, pH 7.2) to an OD_600_ of 0.3. Bacterial suspensions were diluted 1 to 10 into either BHI supplemented with IsoVitaleX at the indicated concentrations or CDM (20 μL into 180 μL per well). Growth was then assayed by OD_600_ reading every 30 min for 32 h using a Tecan Spark microplate reader (Tecan Systems) at 37°C with orbital shaking. Absorbance values were determined using the average of triplicate wells and subtracting the medium background, as determined by the sterility control of medium only.

### Western analysis of O-antigen

Bacteria were resuspended in ~5 mL PBS to achieve an OD_600_ of 0.5 (~1x10^9^ CFU/mL), and 1 mL aliquots were used to prepare whole-cell extracts. Cell pellets were suspended in 1x NuPage gel loading buffer, heated for at least 45 min at 99 to 100°C, and confirmed to be inactivated. Whole cell extracts equivalent to approximately 10^7^ CFU were size-separated on a NuPage Novex 4-12% Bis-Tris gel and transferred to a nitrocellulose membrane using an iBlot Gel Transfer Device. The membranes were blocked with 5% skim milk in Tris-buffered saline containing 0.5% Tween-20. Membranes were probed with mouse monoclonal α-lipopolysaccharide (LPS) antibody (FB11; Thermo Fisher Scientific) or α-capsule antibody (11B7 (Apicella et al., 2010)) at 1:500 dilution and detected using an HRP-conjugated goat polyclonal secondary antibody at 1:5000 dilution. GroEL (approximately 60 kDa) was used as a loading control at a dilution of 1:2000 (Enzo Life Sciences). Bands were visualized using either a Clarity Max ECL or Pierce 1-Step Ultra 3,3’,5,5’-tetramethylbenzidine Blotting Substrate kit following the manufacturer’s instructions. Where indicated, the samples were treated with 200 μg of Proteinase K (Qiagen) for 1 h prior to size separation.

### Intracellular growth analysis

The J774A.1 murine macrophage like cell line (American Type Culture Collection TIB-67) was seeded at 2.5x10^5^ cells per well in 24-well plates and cultured in Dulbecco’s Modified Eagle’s medium (DMEM) with 10% Fetal Bovine Serum for 24 h at 37°C with 5% CO_2_. Bacteria were resuspended in PBS to OD_600_ of 0.2 and diluted 1:5 in DMEM. The bacterial inoculum (200 μL) was added to macrophages (multiplicity of infection of 100:1) and incubated for 2 h. Following this incubation, macrophages were washed 3x with PBS and the culture medium was replaced with DMEM with 25 μg/mL gentamicin. At 4 h and 24 h, wells were washed 3x with PBS and *F. tularensis* was recovered using 200 μL of sterile water to lyse the macrophages. The recovered bacterial cells were immediately serially diluted in PBS and plated on chocolate agar for enumeration. These data shown are the average of at least three independent experiments.

### LD_50_ determination in a murine model

Virulence was assessed using an intranasal challenge in 7–9 week old BALB/c female mice (Charles River Laboratories) using 10 mice per group and five challenge groups for each strain. Challenge doses were prepared using fresh bacterial suspensions in PBS and enumerated on chocolate agar. Mice were anesthetized prior to the challenge using ketamine, acepromazine, and xylazine injected intraperitoneally (~0.15mL per 20 g of body weight). The intranasal challenge was performed using 50 μL inoculum. The mice were monitored at least daily for 14 days, and observations increased to at least twice daily when clinical signs began to occur. Mortality or euthanasia as determined by an endpoint score sheet was recorded to determine the LD_50_ of each strain. When required, euthanasia was performed in accordance with American Veterinary Medical Association guidelines (Leary et al., 2020) using approximately 200 mg/kg of Euthasol^®^ solution.

### Aerosol challenge of Fischer 344 rats

*F. tularensis* challenge material was prepared from supplemented BHI cultures grown overnight in a 37°C shaker at 200 rpm. Cultures were harvested by centrifugation, re-suspended to the concentration yielding the desired challenge doses in fresh BHI and confirmed by serial dilutions and plating (Roy et al., 2003). The range of doses used for the exposures of the rats were as follows: VT68 (113-1.15x10^6^ CFU); Strain 425 (32-1.54x10^5^ CFU); and Strain 503 (141-1.48x10^6^ CFU). Aerosolized doses of the *F. tularensis* strains were administered to the rats (6–8 weeks old, 8 per group) using a dynamic 30-liter humidity-controlled Plexiglas whole-body exposure chamber, as previously described. Aerosol challenges were sampled using an all-glass impinger vessel to calculate the indicated inhaled challenge dose as previously described (Glynn et al., 2005; Dabisch et al., 2012b; Dabisch et al., 2012a). The rats were monitored multiple times each day, and mortality (or euthanasia as described above) was recorded for 21 days to determine the LD_50_ of each strain.

### Pathology

Postmortem tissues (lung, spleen, and liver) from at least two animals in each challenge group were collected from the challenged rats. Briefly, the tissues were fixed in 10% neutral buffered formalin, embedded in paraffin, and sectioned for hematoxylin and eosin (HE) staining. At least one section of the above tissues was examined by a board-certified veterinary pathologist and subjectively graded on the severity of necrosis/inflammation as: minimal (involving *<* 10% of the tissue), mild (involving 11-25% of the tissue), moderate (involving 26-50% of the tissue), marked (involving 51-79% of the tissue), or severe (involving *>* 80% of the tissue).

### Genomic analysis

Each strain had been previously sequenced (VT68: CP010288; 425: CP010289; 503: NZ_LVKX01000000). In this study, USAMRIID repository vials of VT68 and 503 and strains were sequenced on the Illumina MiSeq platform using the Illumina Nextera Flex kit and a 2x150 cycle kit. VT68 and 503 strains were additionally sequenced on the Oxford Nanopore Mk1C platform using the ligation sequencing kit (LSK109) and native barcoding (NBD-104) for library preparation, then run on a version 9 flow cell. The combined short and long read data was assembled through Unicycler (v0.4.8) (Wick et al., 2017), after quality filtering short reads with trimmomatic and long reads with filtlong (Bolger et al., 2014). Reads were mapped back to consensus genomes to assess assembly quality. VT68 had a coverage of 100% and an average depth of 721x with short reads, and a coverage of 100% and an average depth of 304x with long reads. Strain 503 had a coverage of 100% and an average depth of 2,424x with short reads, and a coverage of 100% and an average depth of 64x with long reads. All mappings produced even approximately even coverage distribution, indicating an absence of assembly errors. The 425 (CP010289.1) and LVS (NC_007880.1) sequences were downloaded from GenBank. The assembled genomes were processed using SNPeff (v4.3) (Cingolani et al., 2012) with LVS (Live Vaccine Strain) as the reference strain. To identify common and unique genes, an analysis was performed using Roary (v3.13.0) (Page et al., 2015), with CDS identified and annotated with PROKKA (v1.14.6) (Seemann, 2014). The average Nucleotide Identity (ANI) was calculated following the formula of Goris et al (Goris et al., 2007), as implemented in the Kostas’ lab ANI calculator: Kostas lab | ANI calculator.

### Statistics

For the bacterial growth curve data analysis, a logistic growth curve of each well was fitted as previously described (Zwietering et al., 1990) which yielded estimates of the lag time, maximum growth rate, and asymptote. The area under the curve (AUC) for each well was calculated as previously described (Shiang, 2002). The significance of pairwise strain comparisons was obtained from linear contrasts in the weighted least squares model. For bacterial intracellular replication analysis, pairwise strain comparisons for CFU data were performed using a negative binomial generalized linear model. No multiplicity adjustments were made. Mouse and rat LD_50_ measurements were estimated using a Probit Model with Log transformation of the dose variable. Where a 0 dose is reported, it was inputted to one-tenth of the value of the next highest concentration. The pair-wise comparisons were based on a Probit model, assuming a common slope. The median time to death (TTD) was estimated using the Kaplan-Meier method. Pair-wise comparisons were made using Wald Chi-square tests based on a Cox regression model. The analysis was implemented using SAS version 9.4 (SAS Institute, Inc., Raleigh NC).

## Results

### *In vitro* growth analysis of VT68, 425 and 503

**Figure 1:**
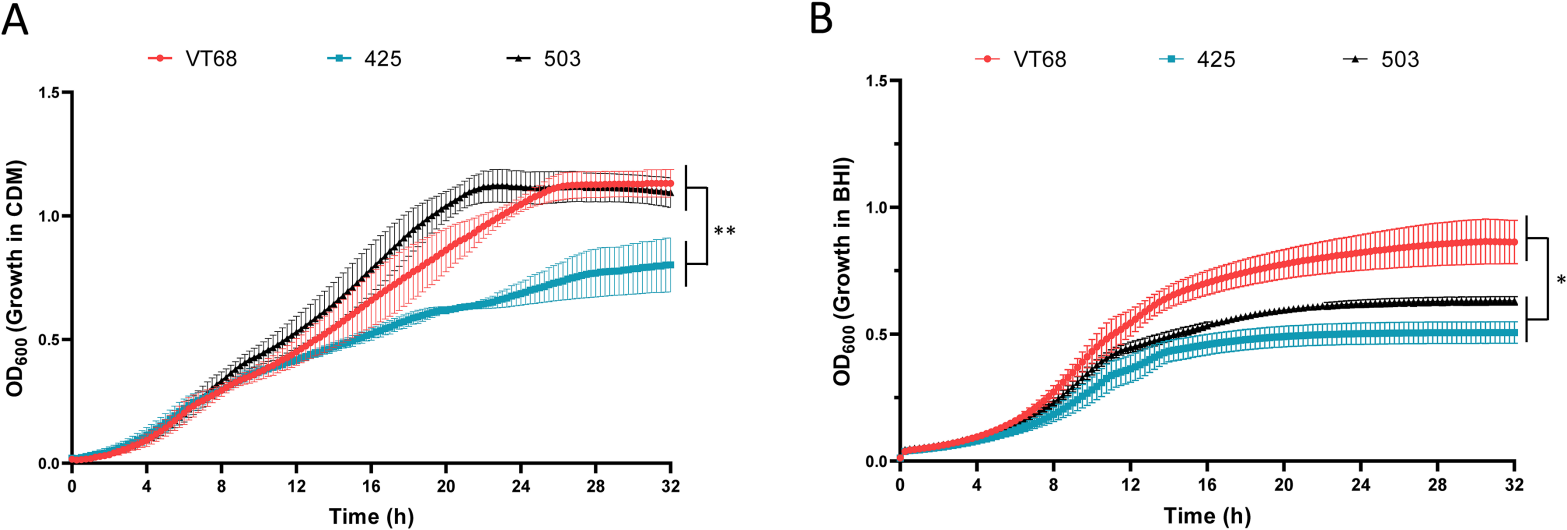
Growth analysis of the historical *F*. *tularensis* isolates VT68, 425, and 503. Each strain was cultured in **(A)** Chamberlains defined medium or **(B)** brain heart infusion medium supplemented with 2% IsoVitaleX shaking at 37°C. Growth was monitored for 32 hours by OD_600_ readings. These data represent the average of three separate experiments. Error bars represent the standard error of the mean. Significance was assessed by pairwise comparisons obtained from linear contrasts in a weighted least squares model. *p<0.05, **p<0.01.

It has been established that the culture medium in which *F. tularensis* is grown can result in an expression profile of proteins and surface features that mimic either a non-host adapted or host adapted state (Hazlett et al., 2008; Holland et al., 2017). For this reason, we examined the planktonic growth of each *F. tularensis* ssp*. holarctica* strain in CDM, a nutritionally rich defined medium, and in BHI + 2% IsoVitaleX, a complex medium that more closely mimics a host adapted state. In agreement with the findings for other *F. tularensis* isolates (Bachert et al., 2021), all strains tested in this study reached a higher cell density as measured by OD_600_ when grown in CDM (**Figure 1A**) compared to supplemented BHI (**Figure 1B**). In CDM, VT68 and 503 were able to obtain a final OD_600_ > 1.0. In contrast, 425 yielded a significantly lower final OD_600_ (~0.6) when grown in CDM (**Figure 1A**, blue squares) than to VT68 (p<0.01) and 503 (p=0.0001).

We previously observed that some *F. tularensis* isolates grew poorly in BHI supplemented with 1% IsoVitaleX (Bachert et al., 2021). Therefore, we tested additional concentrations of IsoVitaleX and found that the final *F. tularensis* culture density increased with additional IsoVitaleX supplementation (**Supplementary Figure S1**). Based on these data, we compared the growth of each isolate in BHI medium with 2% IsoVitaleX as this concentration did not alter the culture lag time (p>0.05) but increased the area under the curve (p<0.05). Under this condition, growth rate (OD_600_/h) of VT68 was significantly faster than that of both 425 (p<0.01) and 503 (p<0.05). In BHI, 425 and 503 did not differ significantly in growth, although the final OD_600_ of 425 was decreased by comparison.

### A unique low molecular weight O-antigen band is found in the LPS of 503, but intracellular replication is maintained

**Figure 2:**
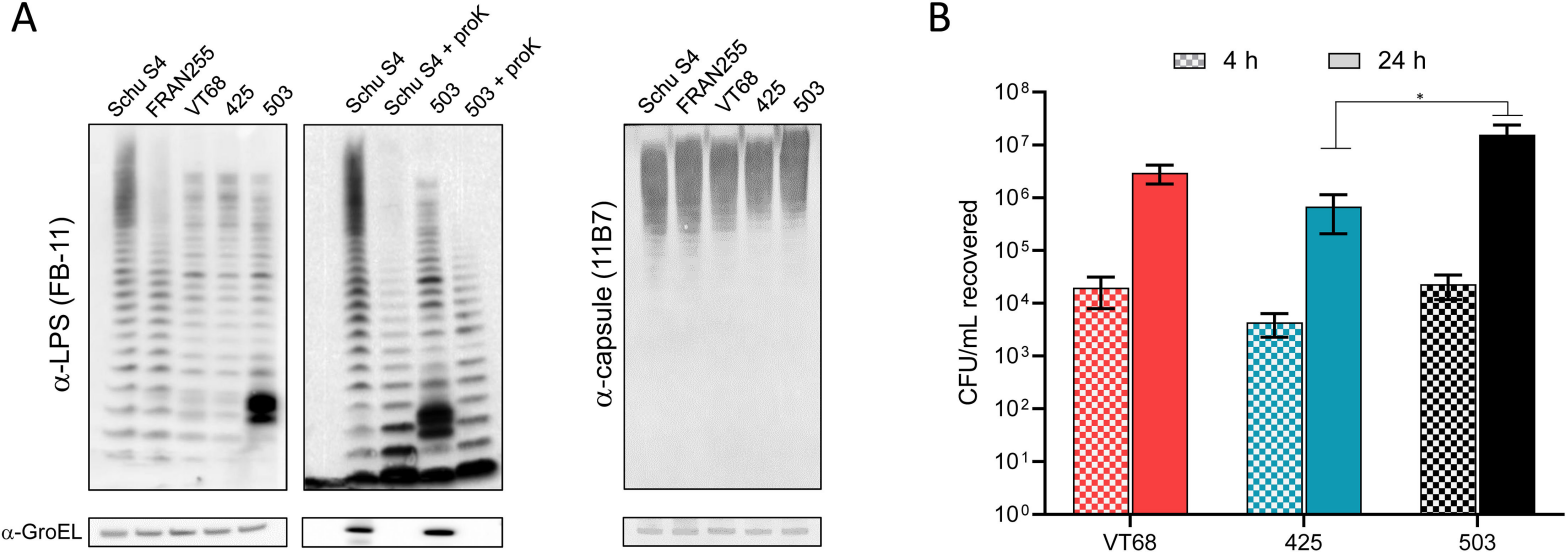
O-antigen profiling and intracellular replication of type B isolates. **(A)** Whole cell extracts at equal concentrations of each strain were separated by SDS-PAGE gels and blotted with either a monoclonal antibody to the O-antigen of LPS (FB11) or capsule (11B7) of *F*. *tularensis*. For analysis, GroEL protein was used as a loading control (approximately 60 kDa). Where indicated, samples were treated with proteinase K (+ proK) prior to loading and blotted with the LPS antibody. **(B)** Intracellular bacterial growth in J774A.1 macrophage-like cells is shown after 4 (checkered) and 24-h (solid) for VT68, 425, and 503. Growth was measured in terms of colony forming unit (CFU) recovered using gentamicin protection assays. Error bars represent the SEM from at least three independent experiments. *p<0.05.

*F. tularensis* LPS and capsule play a critical role in host immune evasion as mutations in the biosynthesis genes encoding for these structures results in attenuation (Raynaud et al., 2007; Weiss et al., 2007; Apicella et al., 2010; Rasmussen et al., 2014; Chance et al., 2017). Whole-cell extracts were prepared and probed using a monoclonal antibody directed at the O-Ag of LPS (**Figure 2A**, left) or capsule (**Figure 2A**, right). The overall banding pattern was similar to that observed in previous studies for both LPS and capsule (Bachert et al., 2021) with the notable exception of 503, which featured dense low molecular-weight banding. To determine whether the banding in 503 was due to the presence of an O-Ag conjugated protein, we treated whole cell extract samples with proteinase K to degrade any associated protein component. After this treatment, the low molecular weight bands were eliminated from the LPS profile of 503 (**Figure 2A****, center**), suggesting the presence of an abundant unknown O-antigen conjugated protein present in 503 but not detected in the other Type B strains under our preparation conditions.

We next assessed the ability of these three Type B isolates to grow intracellularly within the murine macrophage-like J774A.1 cell line using a gentamicin protection assay (**Figure 2B**). This assay confirmed that all isolates were able to infect and replicate within these host cells. At 4 h, similar CFUs were recovered for VT68 and 503 (2.0x10^4^ and 2.3x10^4^ CFU). Recovery for 425 was less than the other isolates (4.3x10^3^ CFU), though this difference was not significant. At 24 h, an increase in CFU of approximately 2 logs was observed for both VT68 and 425. Interestingly, the CFUs recovered at 24 h were greater for 503 (*p*<0.05 compared to 425; *p* = 0.19 compared to VT68), although the percent increase between the timepoints was no different than that of the other strains. Ultimately, each isolate was able to successfully gain entry and replicate within macrophages, with only mild differences in the degree of replication displayed between the three isolates.

### Type B strains are highly lethal in a pneumonic murine model, but 425 displayed mild attenuation and a delay in time to death

**Figure 3:**
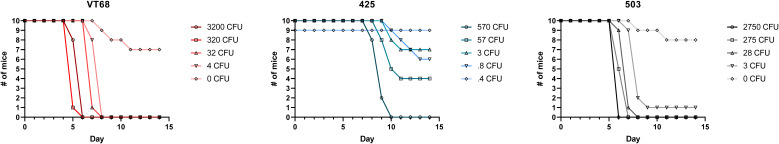
Intranasally challenged mice demonstrate lethality of each Type B isolate, but strain 425 displays a delayed time to death and increased LD_50_. Groups of mice (n=10) were challenged by the intranasal route with VT68, 425, or 503 with varying CFU doses as listed in the legends and monitored for survival for 14 days post challenge. The LD_50_ and TTD values were determined as indicated in **Table 2**. Each data point represents an individual animal. The challenge doses were determined from CFU counts from the serial dilutions on chocolate agar plates. For those challenge doses were less than 1 CFU is listed, this number is based upon the average CFU number calculated.

The virulence of these *F. tularensis* ssp. *holarctica* strains was assessed using a pneumonic murine model. Groups of BALB/c mice were challenged intranasally with five doses of the respective strain and monitored for survival over the course of 14 days to determine the LD_50_ (**Figure 3**; **Table 2**). Both VT68 and 503 were highly virulent in this model, with an LD_50_ of ~1 CFU, which is similar to the results of our other studies with *F. tularensis* strains (Bachert et al., 2021). In contrast, the LD_50_ of 425 was found to be higher (9 CFU). Although the mice were still sensitive to 425 by this challenge method, both the LD_50_ and TTD were significantly different compared to VT68 and 503 (*p*<0.0001). Mice challenged with VT68 and 503 succumbed six days post challenge at the highest challenge dose (10^2–^10^3^ CFU) and eight days at the lowest lethal dose (1–10 CFU). In contrast, 425 exhibited a delay in the TTD relative to the other test strains. The median TTD was nine days post challenge at the highest dose and 10.5 days post challenge at the lowest lethal dose (10–100 CFU; **Table 2**).

**Table 2:** Virulence of Type B isolates in a pneumonic tularemia murine model.

		VT68	425	503
	LD_50_ (CFU)	< 1	9.4	< 1
				
Median TTD (Days)	0.1 - 1 CFU	>14	>14	>14
	1 - 10 CFU	8	>14	8
	10 - 100 CFU	7	10.5	7
	100 - 1000 CFU	5	9	6.5
	1000 - 10,000 CFU	6		6
				
Pair-Wise Comparisons of TTD (Wald-test)	vs. 425	p<0.0001		
	vs. 503	NS	p<0.0001	

NS, Not Significant (P>0.05); TTD, Time to Death.

### Strain 425 is completely attenuated via aerosol exposure in Fischer rat models while VT68 and 503 are virulent

**Figure 4:**
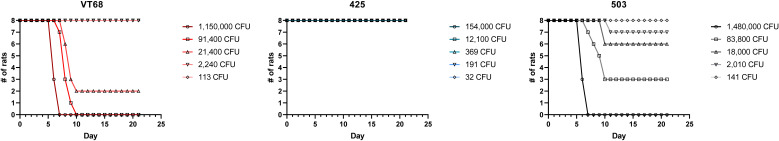
Aerosol challenge of Fischer rats shows attenuation of strain 425 while VT68 and 503 remain virulent. Groups of Fischer rats (n=8) were challenged by whole body aerosolization with VT68, 425, or 503 with varying CFU doses as listed in the legend and monitored for survival for 21 days post challenge. The LD_50_ and TTD values were determined as indicated in **Table 3**. Each data point represents an individual animal.

While the tularemia murine model is sufficient to provide an initial assessment of virulence, it has been suggested that the Fischer rat model is more appropriate for characterization of tularemia pathogenesis and vaccine protection studies (Raymond and Conlan, 2009; Wu et al., 2009; Ray et al., 2010). Rats were challenged via whole-body aerosol exposure, and survival was monitored for 21 days (**Figure 4**; **Table 3**). In aerosol challenged rats, the LD_50_ for VT68 and 503 was determined to be 18,347 and 39,343 CFUs, respectively. The LD_50_ differences between these two Type B strains were not found to be significant. At the highest challenge dose (~10^6^ CFU), both VT68 and 503 displayed a median TTD of six days. Within the LD_50_ range (10^4^ to 10^5^ CFU) the median TTD was nine days for VT68, whereas survivorship (>21 days) would be expected for 503 at this dose.

**Table 3:** LD_50_ analysis of Fischer rats following aerosol exposure to Type B isolates.

		VT68	425	503
	LD_50_ (CFU)	18,347	N/A	39,343
				
Median TTD (Days)	100-1000 CFU	>21	>21	>21
	1000 - 10,000 CFU	>21	>21	>21
	10,000 - 100,000 CFU	9	>21	>21
	100,000-1,000,000 CFUCFU	8	>21	10
	> 1,000,000 CFU	6		6
				
Pair-Wise Comparisons (Wald-test)	vs. 425	p=0.007		
	vs. 503	p=0.014	p=0.040	

TTD, Time to Death.

Unexpectedly, an LD_50_ value of 425 was unable to be determined because no rats succumbed to aerosol challenge even at the highest dose delivered (1.54x10^5^ CFU). Animals challenged with a high dose (10^5^ CFU) of 425 showed mild signs of tularemia (chromodacryorrhea) at 6–7 days post challenge. However, these signs quickly resolved, and no other clinical observations were noted for the remaining time of the study.

### Histopathologic analysis of rats challenged with virulent Type B isolates suggests VT68 and 503 may have different infection dynamics

**Figure 5:**
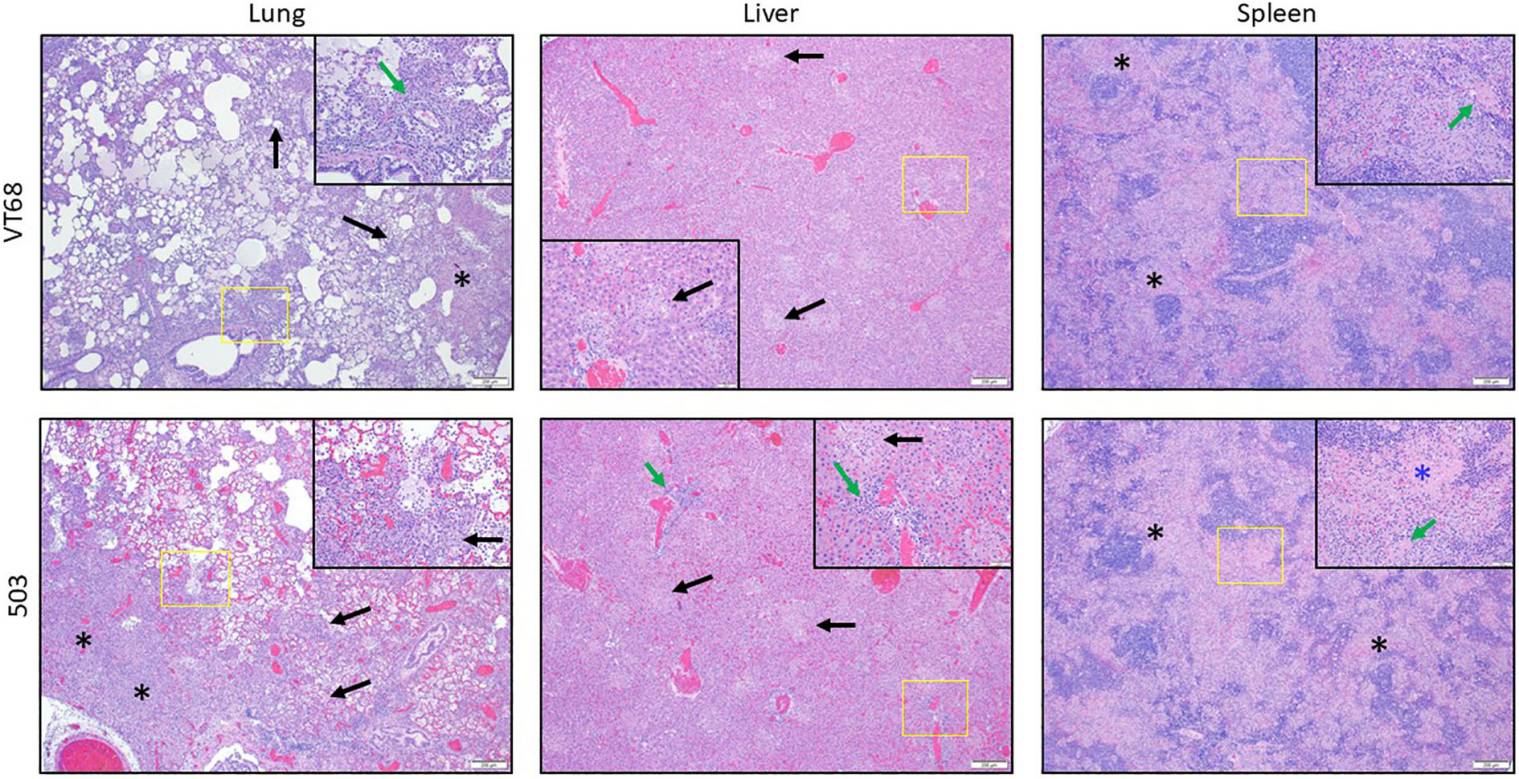
Histopathological analyses from VT68 and 503 aerosolized challenged Fischer rats. Rats were aerosol challenged with VT68 (top) or 503 (bottom) at 1x10 (Kaufmann et al., 1997) calculated inhaled CFU. Rats were necropsied at death or euthanized when meeting intervention criteria. The lungs (left), liver (center), and spleen (right) were examined for histopathology. A representative animal from each group is shown. Lung images - left: In both groups there is neutrophilic and mononuclear alveolar inflammation, necrosis and dense fibrin/edema/debris filling alveoli in a patchy, multifocal pattern (black arrows) (slightly more severe/extensive in group 503), as well as necrosis and inflammation surrounding vessels (green arrow). There are areas of consolidation where adjacent alveoli are entirely filled with fibrin and necrotic debris (black asterisks). Liver images – center: There is multifocal, random, lytic hepatocyte necrosis (black arrows, appears as multifocal areas of pallor) and inflammation in animals from both groups. The areas of necrosis are infiltrated by variable numbers of macrophages and neutrophils. There is hepatocyte vacuolation (left side of image inset) and mononuclear periportal inflammation in the group 503 animal (green arrows). Spleen images – right: There is marked depletion of all compartments of the white pulp in the spleen of animals from both groups, which is most prominent in marginal zone (black asterisks). There is necrosis in both the red and white pulp and accumulation of abundant fibrin, particularly in the marginal zone (prominent in the group 503 image inset – blue asterisk). There are fibrin thrombi present in both image insets (green arrows).

Dissemination studies have shown that the lungs, liver, and spleen are the major target organs affected by pneumonic challenge with *F. tularensis* (Kijek et al., 2019). For rats challenged with VT68 or 503, histopathological analysis was performed on these target organs for at least two animals that succumbed to infection at the highest challenge doses (10^6^ CFU) (**Figure 5**) and those that survived to the end of the study (10^4^ CFU) (**Supplementary Figure S2**). In general, both VT68 and 503 animals challenged with the 10^6^ CFU dose displayed ongoing inflammation and necrosis at the time of death compared to survivors challenged with 10^4^ CFU. In surviving animals, necrosis appeared to be absent, and markers of recovery were observed in most instances (discussed below). Generally, this distinction was present in each target tissue and was similar to what has been previously observed in aerosol challenged Fischer rats (Mlynek et al., 2023).

The lungs of animals challenged with a lethal dose (10^6^ CFU) of VT68 or 503 contained extensive lesions with abundant necrosis and inflammation that appeared in a multifocal to coalescing patchy pattern in most lobes, including regions with locally extensive damage (**Figure 5**, left). The 10^4^ CFU challenged survivors also had inflammatory lesions, but they were often more mononuclear than neutrophilic, and necrosis was absent (**Supplementary Figure S2**, left). In many cases, lesions appeared to represent the subacute phase or an attempt at resolution of damage sustained in the acute phase of disease.

Liver tissue samples from rats infected with 10^6^ CFU of VT68 or 503 displayed lesions that predominantly consisted of lytic necrosis, inflammation, and hepatocyte vacuolation. Lytic necrosis was multifocal and consisted of random clusters of hepatocytes with loss of tissue and, in some cases, cellular architecture (**Figure 5**, center). The liver tissue of survivors challenged with the lower 10^4^ CFU dose of 503 contained only a few lesions of minimal severity, similar to liver tissues from VT68–10^4^ CFU challenged survivors which also had few lesions that, in all cases, were of minimal severity (**Supplementary Figure S2**, center).

Spleen tissue from the 10^6^ CFU challenged rats for both strains showed necrosis in both the red and white pulp as well as signs of lymphoid depletion, inflammation, and accumulation of fibrin in animals challenged with a high dose of either strain (**Figure 5**, right). A marked difference was noted between the tissues of rats receiving 10^4^ CFU (**Supplementary Figure S2**, right) versus 10^6^ CFU (**Figure 5**, right) for both VT68 and 503. Notably, both groups of rats receiving the lower dose showed tissue with limited lesions that were of minimal to mild severity.

### Genomic assessment of VT68, 425, 503

**Figure 6:**
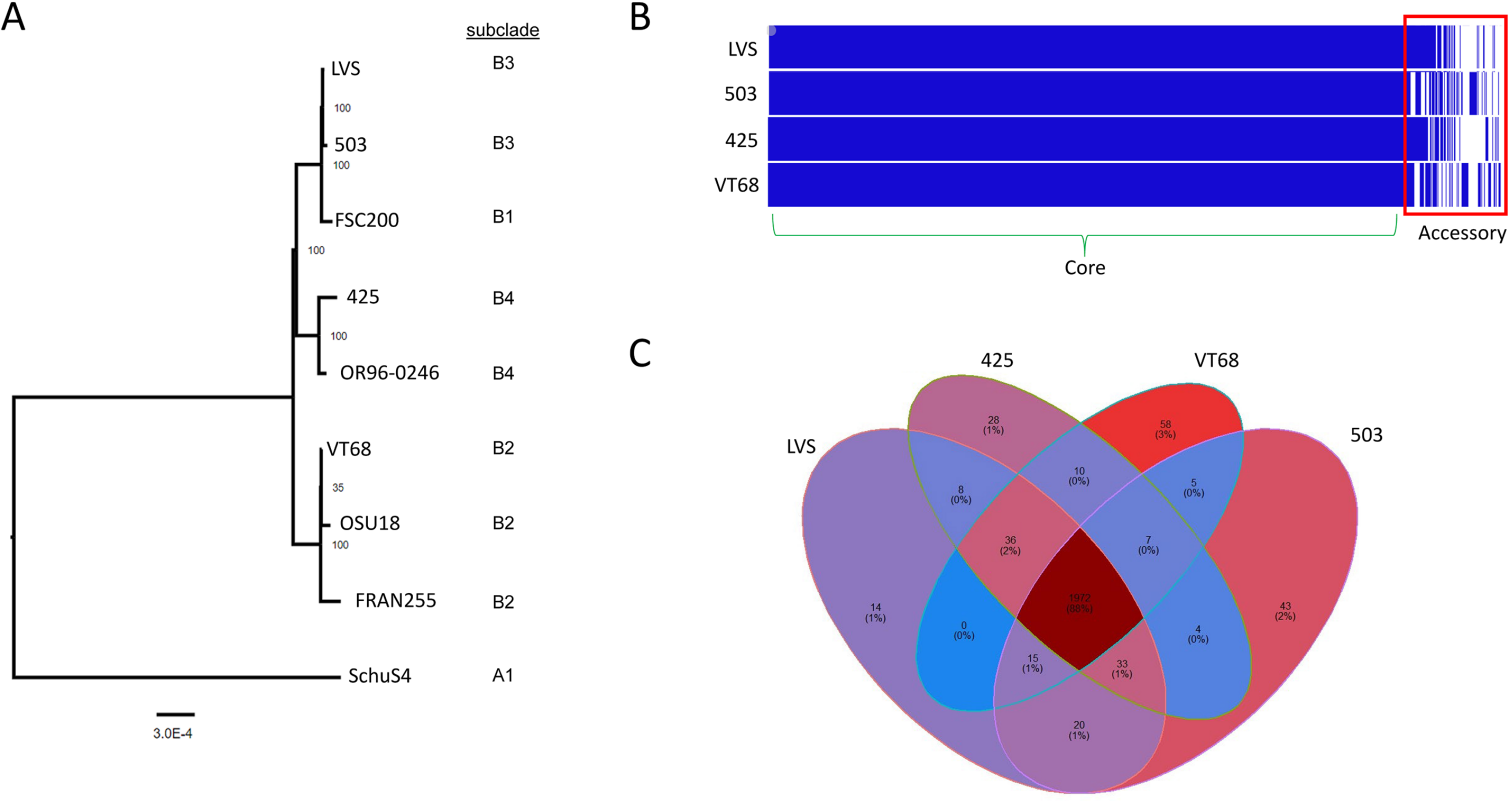
Genomic context and content of VT68, 425 and 503. **(A)** A phylogenetic tree was generated based upon 1,000 genes from each strain listed using the codon tree method provided by the BV-BRC to align proteins and coding DNA from single copy genes using RAxML. Subclade typing was performed manually based on the genome content *post hoc*. **(B)** The presence/absence of all open reading frames was assessed across each genome using Roary and visualized with Phandango. Blue coloring indicates gene presence and white indicates gene absence. Genes shared across all genomes were labeled the core (green parenthesis) and differentially present genes were labeled as accessory (red box). **(C)** The percentages of unique or shared genes for each strain based of Roary analysis among each strain studied.

Each of these Type B isolates was previously sequenced in an effort to bolster the genomic data available to develop diagnostic detection tools (Johnson et al., 2015), although the genomic content and context of these strains remained unassessed. To compare the genomes of these strains relative to each other, a phylogenetic tree based on codon relatedness was constructed using additional Type B isolates for which virulence data are available (LVS, FSC200 (Svensson et al., 2012), OSU18 (Petrosino et al., 2006), OR96-0246 (Atkins et al., 2015) and FRAN255 (Bachert et al., 2021)) as well as SchuS4 (FRAN244 (Bachert et al., 2021)), a prototypical *F. tularensis* isolate of Type A origin (**Figure 6A**). As expected, there was generally a high degree of relatedness observed in the codon tree, with the greatest distance observed between the Type B isolates and Schu S4 (Type A), highlighting that the genomic context of each strain is indeed Type B. Strain 503 clustered most closely to the node containing LVS with FSC200 also included in this subclade. The next most related node contained 425 and OR96-0246, and together these Type B isolates clustered more distantly from VT68, OSU18, and FRAN255. Although extremely limited, this analysis suggests that VT68, 425, and 503 are reasonably diverse from each other, especially given the virulence data available for Type B isolates (Vogler et al., 2009; Bachert et al., 2021). Furthermore, each strain belonged to a different subclade (Svensson et al., 2009).

Though LVS is attenuated and used as a BSL-2 surrogate, it is arguably still the best characterized and annotated Type B isolate available to date. To detect large-scale genomic rearrangements, such as inversions and insertions/deletions >1kb, we performed whole-genome alignment of VT68, 425, and 503 using LVS as a comparator strain. Overall, the strains were generally syntenic with only three inversions discovered comparing each strain to LVS, with each inversion flanked by an ISFTU1 repetitive insertion element (**Supplementary Figure S3**). VT68 and 425 share an approximately 15.9 kb inversion encompassing the corresponding region of FTL_0164 to FTL_0185 in the LVS. A second 12.2 kb inversion was found in 425 spanning FTL_1630 to FTL_1643. Strain 503 contained an approximate 60.1 kb region (FTL_1925 to FTL_0023) that was inverted relative to the LVS; however, no coding sequences were disrupted.

ANI analysis suggests that, at most, the Type B isolates analyzed here differ at most by 0.08%, with 503 differing by only 0.02% when compared to LVS (**Supplementary Table S1**). To isolate gene presence/absence data across these strains, Roary analysis was implemented to delineate the core genome from the accessory genome (**Figure 6B**). This comparison determined that the core genome contained 1,972 genes (88% of the total open reading frames (ORFs) identified) with an accessory genome containing 281 ORFs. In total, 129 unique coding sequences were present with 58 ORFs in VT68, 28 in 425, and 43 in 503 (**Figure 6C**). Perhaps unsurprisingly, many of the observed unique genetic differences identified by Roary are due to variations within ISFtu1/2 transposases. Removing these from the dataset resulted in 68 changes in ORFs relative to the LVS across the three genomes (**Table 4**). Comparison among each individual strain suggested 12 ORFs were uniquely disrupted in 425 while 7 ORFs were uniquely absent. In VT68, there were 4 unique splice events, 4 unique disruptions and 2 unique absences. Strain 503 again contained the fewest alterations with only 3 unique disruptions and 1 absent ORF.

**Table 4:** Unique genetic differences contained in each Type B genome relative to LVS.

Gene	Annotation	Tag	Summary of change relative to LVS		
			425	VT68	503
*scrB*	Sucrose-6-phosphate hydrolase	HDKEJNGD_00059	Single ORF spanning 00059 and 00060	Single ORF spanning 00059 and 00060	
group_28	hypothetical protein	HDKEJNGD_00060			
*dmlR_2*	HTH-type transcriptional regulator DmlR	HDKEJNGD_00071	Single ORF spanning 00071 and 00072	Single ORF spanning 00071 and 00072	Single ORF spanning 00071 and 00072
group_40	hypothetical protein	HDKEJNGD_00072			
group_212	hypothetical protein	HDKEJNGD_00074		Two ORFs spanning 00074-00076	
group_41	hypothetical protein	HDKEJNGD_00075			
group_42	hypothetical protein	HDKEJNGD_00076			
*clcA*	H(+)/Cl(-) exchange transporter ClcA	HDKEJNGD_00112	Single ORF spanning 000112 and 00113	Single ORF spanning 000112 and 00113	Single ORF spanning 000112 and 00113
*clcA_2*	H(+)/Cl(-) exchange transporter ClcA	HDKEJNGD_00113			
group_2249	LpsA protein	HDKEJNGD_00158	Stop lost		
group_809	Acid phosphatase (EC 3.1.3.2)	HDKEJNGD_00182		Stop introduced: Gln314*	
group_54	hypothetical protein	HDKEJNGD_00303		Split into two ORFs	
group_214	hypothetical protein	HDKEJNGD_00311		Truncated ORF	
*phrB*	(6-4) photolyase	HDKEJNGD_00380	Stop introduced Gln61*		
group_58	hypothetical protein	HDKEJNGD_00387	single ORF spanning 00387 and 00388	single ORF spanning 00387 and 00388	
group_59	hypothetical protein	HDKEJNGD_00388			
group_61	hypothetical protein	HDKEJNGD_00399	ORF split in two	ORF split in two	
group_215	hypothetical protein	HDKEJNGD_00402		ORF absent	
group_217	hypothetical protein	HDKEJNGD_00494		ORF absent	
group_218	hypothetical protein	HDKEJNGD_00516	ORF absent	ORF absent	
*thrB_2*	Homoserine kinase	HDKEJNGD_00546	Single ORF spanning 00546 and 00547	Single ORF spanning 00546 and 00547	
*thrB*	Homoserine kinase	HDKEJNGD_00547			
*aguA*	Agmatine deiminase	HDKEJNGD_00554		Stop introduced: Glu223*	
group_75	ATP-dependent helicase HrpA	HDKEJNGD_00651			Stop introduced: Gln894*
*ygaP*	Inner membrane protein YgaP	HDKEJNGD_00697		Stop introduced: Tyr168*	Stop introduced: Gln135*
*dsbB_1*	Disulfide bond formation protein B	HDKEJNGD_00717		Stop introduced: Trp174*	
*panD*	Aspartate 1-decarboxylase	HDKEJNGD_00740	Stop lost	Stop lost	
group_158	hypothetical protein	HDKEJNGD_00854		Stop introduced: Gln51*	
group_219	hypothetical protein	HDKEJNGD_00892	ORF absent		
group_220	hypothetical protein	HDKEJNGD_00898	ORF absent		
group_2102	Bacterial lipoprotein	HDKEJNGD_00922	Stop introduced: Trp90*		
group_168	hypothetical protein	HDKEJNGD_01087	Stop introduced: Gln28*		
*yihQ_1*	Sulfoquinovosidase	HDKEJNGD_01143	Single ORF spanning 01143 and 01144	Single ORF spanning 01143 and 01144	
group_32	Sulfoquinovosidase	HDKEJNGD_01144			
group_1887	PdpC	HDKEJNGD_01262	three ORFs split across 01262		
group_2199	hypothetical protein	HDKEJNGD_01268	two ORFs split across 01268		
group_2110	PdpA	HDKEJNGD_01272	two ORFs split across 01272		
group_222	hypothetical protein	HDKEJNGD_01323	ORF absent		
group_6	hypothetical protein	HDKEJNGD_01365	Stop introduced: Gln204*		
*acsA*	Acetyl-coenzyme A synthetase	HDKEJNGD_01476	ORF absent		
group_224	hypothetical protein	HDKEJNGD_01483			ORF absent
group_2144	hypothetical protein	HDKEJNGD_01604	ORFs absent	ORFs Absent	ORFs absent
group_2154	putative transport protein HsrA	HDKEJNGD_01605			
group_1612	IS5 family transposase ISFtu2	HDKEJNGD_01606			
*ftsH_2*	ATP-dependent zinc metalloprotease FtsH	HDKEJNGD_01607			
group_14	Uncharacterized protein YdiJ	HDKEJNGD_01629	Stop introduced: Glu697*		Stop introduced: Gly815*
group_225	hypothetical protein	HDKEJNGD_01634	ORF absent		
*dacB_2*	hypothetical protein	HDKEJNGD_01649	One ORF spanning 01649 and 01650	One ORF spanning 01649 and 01650	
group_101	hypothetical protein	HDKEJNGD_01650			
*pnuC_2*	Nicotinamide riboside transporter PnuC	HDKEJNGD_01670	Stop introduced: Leu53*		
*ydiK*	Putative transport protein YdiK	HDKEJNGD_01699		one ORF spanning 1699 and 1700	
group_17	hypothetical protein	HDKEJNGD_01700			
group_104	N(4)-(Beta-N-acetylglucosaminyl)-L-asparaginase	HDKEJNGD_01705	One ORF spanning 01705 and 01706	One ORF spanning 01705 and 01706	
group_105	hypothetical protein	HDKEJNGD_01706			
group_35	Cardiolipin synthase	HDKEJNGD_01714		Stop introduced: Trp186*	
group_106	hypothetical protein	HDKEJNGD_01717			Stop introduced: Trp55*
group_198	Bacterial lipoprotein	HDKEJNGD_01726			Stop Lost
group_11	hypothetical protein	HDKEJNGD_01734	Two ORFs split across 01734		
group_112	hypothetical protein	HDKEJNGD_01887	Single ORF spanning 01887 and 01888	Single ORF spanning 01887 and 01888	
group_113	hypothetical protein	HDKEJNGD_01888			
group_20	hypothetical protein	HDKEJNGD_01943	ORF absent	ORF absent	
group_116	Phosphatidylcholine-sterol acyltransferase	HDKEJNGD_02006	Single ORF spanning 02006 and 02007	Single ORF spanning 02006 and 02007	
group_117	Phosphatidylcholine-sterol acyltransferase	HDKEJNGD_02007			
group_118	hypothetical protein	HDKEJNGD_02017	Stop introduced: Gln42*		
group_227	hypothetical protein	HDKEJNGD_02040	ORF absent		
group_228	hypothetical protein	HDKEJNGD_02044	ORF absent		
kdpD_2	Sensor protein KdpD	HDKEJNGD_02049	Stop introduced: Gln145*		
*leuB*	3-isopropylmalate dehydrogenase	HDKEJNGD_02058	Stop lost	Stop lost	

To identify mutations within individual genes that were shared among each strain, we performed single nucleotide polymorphism (SNP) analysis for each genome once again using LVS as the comparator strain. The predicted effect of each mutation was ranked as high (frameshifts/nonsense mutations), moderate (missense mutations), low (synonymous mutations), or potential modifiers (occurring outside the coding region). Overall, 1,722 SNPs were identified with 48 SNPs considered high impact (**Table 5**, **Supplementary Table S2**). In agreement with our phylogenetic codon tree (**Figure 6A**), 503 featured the fewest number of SNPs when compared to LVS (105 total), with only five mutations considered to have a high impact. The more distantly related genomes of VT68 and 425 contained 782 SNPs (23 high impact) and 836 SNPs (20 high impact), respectively, relative to LVS. Most of these alterations affect hypothetical gene products while those of known function that were identified include *phrB*, *pdpC*, *iglF*, *pdpA*, *pnuC*, and *kdpD* in 425; *clcA* and a homolog of *hrpA* in VT68.

**Table 5:** Predicted high impact mutations identified during SNP analysis of Type B isolates.

Strain	Variant Type	REFSEQ Locus Tag	Gene Name	Gene Start	Gene End	Strand	Nucleotide change	Protein change	Variant AA length
VT68	stop gained	FTL_0158	Acid phosphatase (EC 3.1.3.2)	162734	164209	+	c.940C>T	Gln314*	314/491
VT68	stop gained	FTL_0271	Gll0911 protein	257786	258376	+	c.312G>A	Trp104*	104/196
VT68	stop lost	FTL_0320	L-aspartate-beta-decarboxylase	305982	307499	–	c.1516T>C	Ter506Glnext*?	506/505
VT68	stop lost	FTL_0323	Pseudogene				c.737A>C	Ter246Serext*?	246/245
VT68	stop lost	FTL_0348	Pseudogene				c.562T>G	Ter188Gluext*?	188/187
VT68	stop gained	FTL_0467	hypothetical protein	442569	442838	–	c.123G>A	Trp41*	41/89
VT68	stop lost	FTL_0468	Mobile element protein	442789	443469	–	c.654A>G	Ter218Trpext*?	218/217
VT68	stop gained	FTL_0502	Agmatine deiminase (EC 3.5.3.12)	487684	488544	+	c.667G>T	Glu223*	223/286
VT68	stop gained	FTL_0633	Rhodanese-related sulfurtransferases	620610	621128	+	c.504C>A	Tyr168*	168/172
VT68	stop gained	FTL_0652	Putative inner membrane protein	635603	636139	–	c.521G>A	Trp174*	174/178
VT68	stop lost	FTL_0672	Aspartate 1-decarboxylase (EC 4.1.1.11)	659991	660266	–	c.274T>C	Ter92Glnext*?	92/91
VT68	stop gained	FTL_0779	Pseudogene				c.574C>T	Arg192*	192/225
VT68	stop gained	FTL_0780	Cold shock protein of CSP family/hypothetical domain	766774	767205	+	c.151C>T	Gln51*	51/143
VT68	stop gained	FTL_0794	Pseudogene				c.211C>T	Gln71*	71/236
VT68	stop gained	FTL_0819	Pseudogene				c.110G>A	Trp37*	37/133
VT68	stop gained	FTL_0913	Pseudogene				c.40G>T	Glu14*	14/404
VT68	start lost	FTL_1006	Pseudogene				c.2T>C	Met1?	1/114
VT68	stop gained	FTL_1175	Pseudogene				c.190G>T	Gly64*	64/281
VT68	stop lost	FTL_1291	Pseudogene				c.316T>C	Ter106Glnext*?	106/105
VT68	stop gained	FTL_1382	Pseudogene				c.512G>A	Trp171*	171/187
VT68	stop gained	FTL_1570	Phospholipase D family protein	1497910	1499127	+	c.558G>A	Trp186*	186/405
VT68	stop gained	FTL_1631	hypothetical protein	1561074	1562108	–	c.580C>T	Gln194*	194/344
VT68	stop lost	FTL_1887	3-isopropylmalate dehydrogenase (EC 1.1.1.85)	1819695	1820045	–	c.411A>C	Ter137Tyrext*?	137/136
425	stop lost	FTL_0137	LpsA protein	143094	144062	–	c.969G>T	Ter323Tyrext*?	323/322
425	stop lost	FTL_0320	L-aspartate-beta-decarboxylase	305982	307499	–	c.1516T>C	Ter506Glnext*?	506/505
425	stop lost	FTL_0323	Pseudogene				c.737A>C	Ter246Serext*?	246/245
425	stop gained	FTL_0343	hypothetical protein	323554	325083	+	c.181C>T	Gln61*	61/509
425	stop lost	FTL_0348	Pseudogene				c.562T>G	Ter188Gluext*?	188/187
425	stop lost	FTL_0468	Mobile element protein	442789	443469	–	c.654A>G	Ter218Trpext*?	218/217
425	stop gained	FTL_0633	Rhodanese-related sulfurtransferases	620610	621128	+	c.403C>T	Gln135*	135/172
425	stop lost	FTL_0672	Aspartate 1-decarboxylase (EC 4.1.1.11)	659991	660266	–	c.274T>C	Ter92Glnext*?	92/91
425	stop gained	FTL_0844	Transcriptional regulator	825879	826442	–	c.269G>A	Trp90*	90/201
425	stop gained	FTL_1001	hypothetical protein	966911	967522	+	c.82C>T	Gln28*	28/203
425	start lost	FTL_1006	Pseudogene				c.2T>C	Met1?	1/114
425	stop gained	FTL_1250	Mobile element protein	1194482	1195174	+	c.610C>T	Gln204*	204/221
425	stop lost	FTL_1291	Pseudogene				c.316T>C	Ter106Glnext*?	106/105
425	stop gained	FTL_1356	Pseudogene				c.43C>T	Gln15*	15/147
425	stop gained	FTL_1380	Pseudogene				c.217A>T	Lys73*	73/80
425	stop gained	FTL_1489	Uncharacterized protein YdiJ	1414446	1417481	–	c.2089G>T	Glu697*	697/1011
425	stop gained	FTL_1529	Ribosyl nicotinamide transporter, PnuC-like	1459525	1460289	–	c.158T>A	Leu53*	53/254
425	stop gained	FTL_1849	hypothetical protein	1780702	1781280	–	c.124C>T	Gln42*	42/192
425	stop gained	FTL_1879	Osmosensitive K+ channel histidine kinase KdpD	1811748	1812761	–	c.433C>T	Gln145*	145/337
425	stop lost	FTL_1887	3-isopropylmalate dehydrogenase (EC 1.1.1.85)	1819695	1820045	–	c.411A>C	Ter137Tyrext*?	137/136
503	stop gained	FTL_0590	ATP-dependent helicase HrpA	572637	576971	+	c.2680C>T	Gln894*	894/1444
503	stop lost	FTL_1291	Pseudogene				c.316T>C	Ter106Glnext*?	106/105
503	stop gained	FTL_1489	Uncharacterized protein YdiJ	1414446	1417481	–	c.2443G>T	Gly815*	815/1011
503	stop gained	FTL_1573	hypothetical protein	1501135	1501824	+	c.165G>A	Trp55*	55/229
503	stop lost	FTL_1581	hypothetical protein	1507913	1508728	+	c.816G>T	Ter272Tyrext*?	272/271

## Discussion

The goal of this project is to better examine and characterize the Type B strains of *F. tularensis* ssp. *holarctica* since this subspecies is generally not well studied for development and testing of medical countermeasures since they are typically regarded as less virulent. We previously developed a panel of *F. tularensis* strains for use in vaccine testing (Bachert et al., 2021); however, one shortfall of this panel was that it only contained one Type B strain, FRAN255. During a vaccine study implementing our *F. tularensis* diversity strain panel (Bachert et al., 2021) using the rLVS *capB*/*iglABC* vaccine (Jia et al., 2016; Jia et al., 2018), vaccinated rats challenged by whole body aerosolization with strains from the panel were afforded protection; however, when challenged with the Type B strain FRAN255, all vaccinated rats (rLVS *capB/iglABC* or LVS parent) displayed some low-level signs of infection (chromodacryorrhea and weight loss). In addition, one rat in the rLVS *capB/iglABC* single vaccinated group succumbed to infection by FRAN255. In contrast to vaccinated rats challenged with Type A strains, no apparent clinical signs and complete survival was achieved. As the goal of this program was to protect personnel not only to survive exposure but to also enable them to maintain combat readiness, we questioned whether the results observed with vaccinated rats were specific to strain FRAN255 or whether this would be an issue for challenge with any Type B isolate. Therefore, we have begun to characterize additional Type B strains to expand this panel for future vaccine testing.

In the present study, we focused on three Type B isolates (VT68, 425, and 503) that were available at the Biodefense Reference Material Repository, had genomic sequence available and were previously analyzed at USAMRIID or Fort Detrick in various animal models of tularemia. Each of these strains is important from a historical perspective, as VT68 represents a North American Type B isolate obtained from a usual outbreak source, while 425 (USA) and 503 (Russia) share a similar isolation and passage history but were separated geographically. Purposely, each isolate in this study belonged to a different subclade of Type B with other representations (OSU18, OR96-0246, and FSC200) in the literature. In our analysis of these three Type B strains, we characterized them for 1) growth in medium and intracellularly in macrophage-like cells; 2) virulence and pathogenesis in two rodent models of tularemia (BALB/c mice and Fischer rats); and 3) differences when comparing genomic sequences.

When comparing the growth of these strains through the various analyses, 425 did not reach the same maximum optical density as the other two strains when grown in CDM. Likewise, when grown intracellularly in J774A.1 cells, all three strains replicated; however, once again, 425 did not reach a recovered CFU count as high as the other two strains. Initially, we did not expect these two observations to indicate that 425 would be attenuated, as it was previously shown to be virulent in several other animal models (Bell et al., 1955; Owen et al., 1964; Schricker et al., 1972; Fritz et al., 2014). In addition, when BALB/c mice were intranasally challenged with these strains, an LD_50_ value was able to be obtained for all three strains; however, the value for 425 was found to be 9 CFU versus 1 CFU for VT68 and 503. A previous study performed at USAMRIID also using *F. tularensis* strain 425 determined the aerosol LD_50_ in BALB/c mice to be 102 CFU (Fritz et al., 2014). With this determination, it is difficult to draw a direct comparison between the two LD_50_ values with the same strain by different pneumonic challenge routes (small particle aerosol versus intranasal). Likewise, a study by Schricker determined the LD_50_ for 425 by subcutaneous challenge in mice (specific mouse strain not listed) to be 1 CFU (Schricker et al., 1972). Therefore, we expanded these virulence studies to include an additional small animal tularemia model.

We and others have shown that the LD_50_ in Fischer 344 rats aerosol challenged with Schu S4 (Type A) is <500 CFU (Wu et al., 2009; Ray et al., 2010; Mlynek et al., 2023). The Type B isolate OR96–0246 was also tested in a Fischer 344 rat model using intratracheal challenge, which found that the LD_50_ was 10^5^ CFU (Ray et al., 2010; Hutt et al., 2017). These data further support that the Fischer rat model more closely represents human infections than mice as Type B isolates are generally regarded as less virulent than Type A in humans. We previously demonstrated that FRAN255 (Type B; USA isolate) displayed an LD_50_ of 5,672 CFU using whole-body aerosol exposure in rats (Mlynek et al., 2023), but it was unclear how representative this isolate was for Type B isolates in general. Based on the current study, FRAN255 differs from VT68 and 503 in that the LD_50_ value is approximately 10-fold lower by comparison. Genomic analysis also found that FRAN255 clustered differently from VT68, despite both isolates belonging to the B2 subclade of *F. tularensis* ssp. *holarctica* isolates. However, it is currently unclear what genomic variances could account for clustering and virulence phenotypes. Extending virulence studies to aerosol challenged Fischer rats, we found that none of the rats challenged with 425 succumbed to infection, even at the highest challenge dose (1.54x10^5^ CFU). However, some low-level clinical symptoms (chromodacryorrhea) were observed in rats exposed to the 10^5^ CFU dose. In contrast, when rats were aerosolized with VT68 or 503, the LD_50_ values were 1.8x10^4^ and 3.93x10^4^ CFU, respectively. Finding 425 to be attenuated in the rat model was unexpected as it was previously found to be virulent in nonhuman primates (rhesus macaques) and guinea pigs by aerosol exposure in much earlier published studies (Bell et al., 1955; Schricker et al., 1972; Hall et al., 1973). Strain 425 was not found to be virulent in challenged rabbits (Bell et al., 1955; Schricker et al., 1972); however attenuation of Type B strains is typical in this animal species (Brown et al., 2015b; Brown et al., 2015a). To our knowledge, this is the first study to challenge Fischer rats with strain 425.

Genomic sequencing using LVS as a Type B comparator revealed a premature stop introduced within *kdpD* (FTL_1879) at 1013 bp. The *kdpD* gene is 2683 bp and encodes a membrane-bound histidine kinase that phosphorylates a response regulator (KdpE). The KdpD/KdpE two-component system (TCS) is involved as an adaptive regulator necessary for virulence and intracellular survival in several bacterial pathogens (Freeman et al., 2013). However, *F. tularensis* lacks many of the known TCS regulators, including the gene encoding KdpE. Using the surrogate strain *F. novicida*, it was shown that the response regulator PmrA is phosphorylated by KdpD at the aspartic acid located at position 51. Once phosphorylated, it can bind to target promoters and affect gene regulation within the *Francisella* Pathogenicity Island (FPI) (Bell et al., 2010). This TCS in *F. novicida* has been shown to be critical for intracellular replication in macrophages and virulence in mouse models of infection (Mohapatra et al., 2007; Bell et al., 2010). Likewise, in the screening of a *F. novicida* transposon library, *kdpD* was found to be required for virulence in challenged mice and *Drosophila melanogaster* (Weiss et al., 2007; Moule et al., 2010). Most likely, the mutation of *kdpD* in strain 425 leads to its attenuation in both the mouse and rat pneumonic models employed in this study. However, future efforts would need to be performed to verify the role in virulence of KdpD by complementing this version of strain 425 *in trans* with a functional gene on a plasmid and repeating the aerosol challenge with Fischer rats with this new construct to determine if virulence is restored.

However, without this experimental data, we cannot rule out another mutation within strain 425 that could contribute to the observed attenuation. It is difficult to predict the exact effect of a missense mutation on protein function. Our analysis identified 453 missense mutations contained within the 425 genome relative to LVS. It was for this reason we chose to focus on those mutations that would most likely completely disrupt the coding sequence. From this perspective, 19 ORFs were uniquely affected in 425. While we cannot speculate on the effects of deletions on the hypothetical proteins, we noted that the ORFs of *phrB*, *lpsA*, *pdpC*, *iglF*, *pdpA*, *pnuC*, and *kdpD* were disrupted. Of these, *phrB* likely encodes a homolog that has been shown to be involved with DNA repair or maintanence (Dorrell et al., 1993; Cahoon et al., 2011; Graf et al., 2015). The mutation of *pnuC* probably also does not account for complete loss of virulence as this gene appears to be a homolog of a nicotinamide mononucleotide transport component (Zhu et al., 1989; Sauer et al., 2004). Neither *phrB* nor *pnuC* were identified as essential for virulence in a transposon based sequencing approach utilizing Fischer 344 rats (Ireland et al., 2019). From **Tables 4** and **5**, the genetic differences noted for 425, the gene encoding for LpsA protein has a stop loss, which may detrimentally affect protein function or lead to a unique fusion protein. This protein is listed as lipopolysaccharide protein belonging to a glycosyltransferase family. The importance of the O-antigen component of LPS and capsule to *F. tularensis* virulence is well documented in the literature (Raynaud et al., 2007; Apicella et al., 2010; Kim et al., 2012; Jones et al., 2014; Rasmussen et al., 2014; Rasmussen et al., 2015; Chance et al., 2017) and yet, by western blotting extracts of 425, the O-Ag appears to be intact for both the LPS and capsule profiles (**Figure 2A**). Thus, alteration of the *lpsA* gene would not seem to be responsible for the loss of virulence with the 425 strain. A case can be made that three FPI genes (*pdpC*, *iglF*, *pdpA*) may cause attenuation, but due to duplication of the FPI we reason this is likely not the sole reason for attenuation as we have previously observed Type A strains that remain virulent despite mutations in one copy of the gene in question (Bachert et al., 2021). For instance, a frameshift in *pdpC* and *iglF* was identified FRAN251 while an insertion in one copy of *pdpA* was found in FRAN249, a Schu S4 derivative. Both strains retained virulence in mice as the LD_50_ was found to be 1 CFU. Of note, the Type A Coll (FRAN037) strain (Downs et al., 1947) was found to be completely attenuated in a mouse intranasal challenge. Sequencing of this strain identified frameshift mutations within the *pdpB1* gene within the FPI (which encodes a component of the Type VI secretion system) (Bachert et al., 2021). While we cannot rule out the impact of mutations within a single copy or Type A/B differences between the requirement of duplicated FPI genes for full virulence, we hypothesized a different mutation may be more impactful, or the resulting attenuation is due to additive effects involving multiple mutations.

The identification of 425 as attenuated also demonstrates a second case in which historical virulent *F. tularensis* strains have attempted to be used for developing a diverse challenge panel, but the strain has been determined to be attenuated. Modeling in both mice and rats demonstrated attenuation of 425, and we hypothesize the same mutation likely affected virulence in both models. We cannot rule out that the stock used in this study obtained from the BRMR had not experienced genetic alterations from the previously described 425 strain due to the historical methods used in passaging and/or preparation for long term storage of *F. tularensis*. Future efforts could be pursued to determine if stocks exist that are closer to the original 425 strain available from the primary source (Rocky Mountain Laboratory) or those initially provided to the former United States Army Biological Defense Research Laboratory. If such strains were to exist, a genetic comparison could be made between the mutations identified in our current version in this study (425/USAMRIID vial FRAN029) to the formerly used strain. Such a study could determine the status of the *kdpD* gene (and other mutations described here) in relation to when previous animal studies were performed.

One interesting LPS phenotype noted in this strain characterization was with 503. The LPS profile displayed two very large dense bands in the lower molecular weight range, which appeared unique to 503 when compared to the other strains tested. This dense banding was similar to the profile shown for several other *F. tularensis* strains and Schu S4 O-Ag (*waaY* and *waaL*) transposon mutants (Jones et al., 2014). It was noted that this pattern was due to O-Ag glycoproteins, which could be resolved by treatment with proteinase K. Similarly, treatment of extracts from 503 was able to revert the O-Ag profile typically observed for the other strains and demonstrated protein glycosylation with O-Ag. The exact role and importance of protein O-Ag glycosylation in virulent strains of *F. tularensis* remains to be determined, but it is speculated that it may be involved in virulence or assist in combating host immune responses (Jones et al., 2014). Our results with 503 further provide evidence of an additional virulent *F. tularensis* strain demonstrating this O-Ag glycosylation (Balonova et al., 2012; Jones et al., 2014).

Interestingly, the glycosylation effect of the O-Ag profile when using the LPS antibody was observed only in strain 503. When comparing the rat aerosol LD_50_ between the two virulent strains described here, no statistical difference was noted between VT68 (18,347 CFU) and 503 (39,343 CFU) (**Table 3**). If anything, the general trend with these two measurements would be that 503 is less virulent. However, from the histopathologic analysis of rats challenged with these strains, the findings may suggest that the 503 strain is potentially more debilitating than the VT68 strain or that other infection dynamics play a role in lesion severity and character differences observed between the two strains, particularly at higher challenge doses (**Figure 5**).

*F. tularensis* genomes are known to be highly conserved (>99%) as we have previously demonstrated comparing genomic content using our original vaccine test panel which consisted primarily of Type A strains (Bachert et al., 2021). Therefore, for this current comparison with Type B strains only minor genetic differences among our Type B strains were expected (Vogler et al., 2009; Bachert et al., 2021). While historical strains may not be representative of currently circulating Type B isolates, this subspecies is suspected to be highly clonal, suggesting that the analysis of historic strains can be applied to future threats (Farlow et al., 2005; Svensson et al., 2005; Vogler et al., 2009). Our comprehensive genomic analysis supports this observation as we found relatively few differences in the presence or absence of ORFs across these three isolates (<70 genes), many of which were also identified in the SNP analysis, consistent with the introduction or loss of a stop codon to alter ORFs. Excluding ORF variation that mapped to ISFTu 1/2 elements (~45 alterations in total), we observed 29 nonsense mutations, 17 splice variants, and 2 start loss mutations. It did not escape our attention that using LVS as a comparator should have flagged the mutations thought to be responsible for its attenuation (Rohmer et al., 2006; Salomonsson et al., 2009), and our analysis supports these earlier studies. Interestingly, 503 contained four SNPs shared with LVS (FTL_0039, FTL_1246, FTL_1517, and FTL_1521) that were included as class A candidates to explain attenuation, suggesting a high relatedness between these strains. To our knowledge, strain 503 is the closest virulent relative to the LVS and was gifted to the United States for use as a challenge strain to test a production batch of the vaccine for efficacy (Tigertt, 1962).

Of the differences in ORFs present across each of these three Type B strains, nearly half encode hypothetical proteins which prevents defining a function and hypothesizing phenotypic differences. The ORFs encoding DmlR and ClcA are conserved among these three Type B isolates but were flagged in our analysis due to differences compared to LVS. ClcA is a H^+^/Cl^-^ transporter that could help facilitate acid-dependent responses that has been shown to aid intracellular replication within macrophages in Type B isolates (Cakar et al., 2018; Matz, 2021). Further, *clcA* was another mutation proposed to contribute to attenuation in LVS (Rohmer et al., 2006). Other differences include disruption of LpsA, PhrB, and PnuC in strain 425 as discussed earlier (**Table 4**). A nonsense mutation was identified in the ORF encoding HrpA in strain 503. While RNA helicases are known to control processes that contribute to virulence in other bacteria (Salman-Dilgimen et al., 2013; Netterling et al., 2016), it is believed that *HrpA* is pseudogene in *F. tularensis* (Champion et al., 2009) and ultimately the 503 isolate remains virulent. However, it is important to note that without further mutational studies it is difficult to accurately define the potential implications of the differences identified in these isolates relative to LVS.

Currently, the United States biodefense community lacks an approved Food and Drug Administration vaccine to prevent tularemia. As personnel serve in areas where tularemia is endemic or may be a target for the purposeful release of *F. tularensis*, it is important to not only protect them, but also still allow them to perform their mission. As stated above, in a recent study where our *F. tularensis* panel strain was used in a vaccine study, it was noted that rats challenged with a Type B strain (FRAN255) still displayed clinical signs. No clinical signs were noted in vaccinated rats challenged with Type A *F. tularensis*. This study highlighted the need for tularemia vaccine testing to include challenges beyond the Schu S4 strain and a potential issue with protection against Type B strains. As new tularemia vaccines are developed, the characterization of these historical Type B strains will allow the expansion of our panel of *F. tularensis* strains for future efficacy testing. Furthermore, the inclusion of additional Type B strains from recent tularemia outbreaks from diverse geographical areas should also be considered to expand the panel (Dryselius et al., 2019; Esmaeili et al., 2021; Pareek et al., 2025).

## Data Availability

The datasets presented in this study can be found in online repositories. The names of the repository/repositories and accession number(s) can be found in the article/**Supplementary Material**.
