# Supplementary material for: Virulence and genome analysis of three historical Francisella tularensis ssp. holarctica isolates for development of a Type B test panel
Source: Front Cell Infect Microbiol. 2025 Dec 3;15:1679606. doi: 10.3389/fcimb.2025.1679606 (PMC12713356; doi:10.3389/fcimb.2025.1679606)
Supplement: Supplementary file 1 [file DataSheet1.pdf]

Virulence and genome analysis of three historical *Francisella tularensis* ssp *holarctica* isolates for development of a Type B test panel.

Kevin D. Mlynek[1], Joshua B. Richardson[2], Elsie E. Martinez[1], Ronald G. Toothman[1], Ju Qiu[3], Curtis R. Cline[4], Joel A. Bozue[1]

[1]Bacteriology Division, [2]Center for Genome Sciences, [3]Regulated Research Administration Division, [4]Pathology Division, U.S. Army Medical Research Institute of Infectious Diseases (USAMRIID), Frederick, MD, USA

Supplementary Materials

FIGURES:

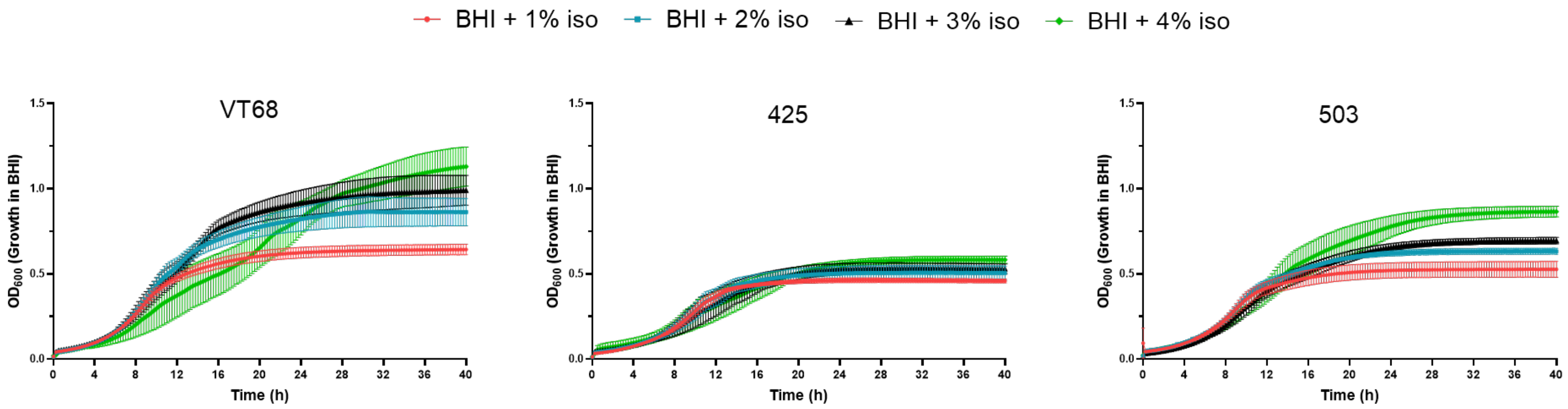

Figure S1. Growth curve analysis of Type B isolates in BHI supplemented with a titration of IsoVitaleX. Each strain was cultured in brain heart infusion medium (BHI) supplemented with the indicated amount of IsoVitaleX (iso) with shaking at 37°C. Growth was monitored for 32 hours by OD_{600} readings. These data represent the average of three separate experiments. Error bars represent the standard error of the mean.

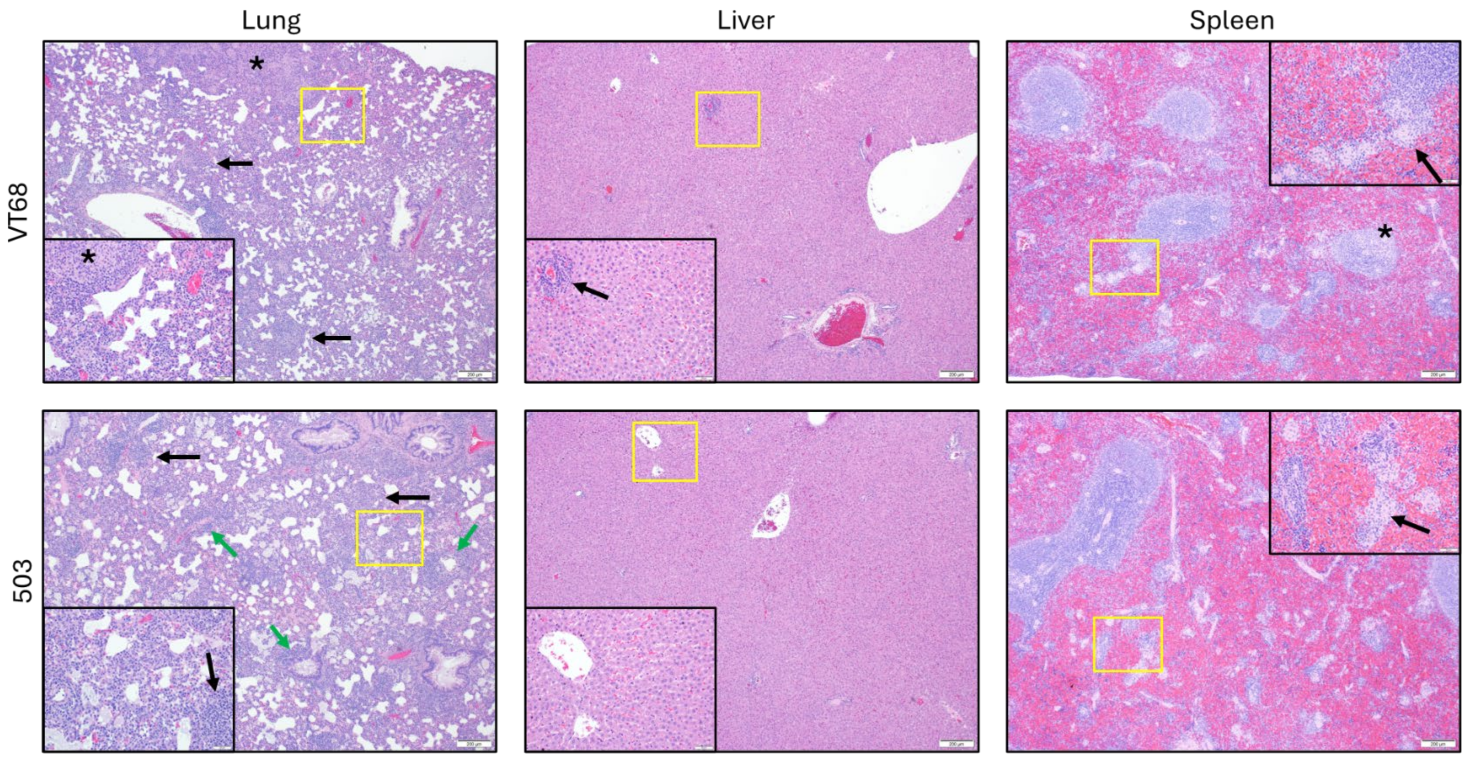

Figure S2. Histopathological analysis of VT68 and 503 at an intermediate challenge dose. Rats were aerosol challenged with VT68 (top) or 503 (bottom) at ~10^4 calculated inhaled CFU. Rats were necropsied at death or euthanized when meeting intervention criteria. The lungs, liver, and spleen were examined for histopathology. A representative animal from each group is shown. Lung images – left: In both groups there are multifocal areas of alveolar inflammation composed predominantly of macrophages, expanding the alveolar septa and filling the lumen (**black arrows**); low numbers of neutrophils are also present in the lung from the group 503 animal. Perivascular and peribronchiolar mononuclear inflammation are present in the group 503 animal (**green arrows**). A dense area of histiocytic inflammation is present in the upper aspect of the group VT68 image, imparting a consolidated appearance to the lung (**black asterisks**). Liver images – center: In the group VT68 image(s) there are few focal areas of mononuclear inflammation (predominantly lymphocytes) in portal areas (**black arrow**), otherwise the liver appears essential normal. The liver image from group 503 is essentially normal. Spleen images – right: There are multifocal areas of mild histiocytic inflammation and/or foci of histiocytic infiltrate in both groups (**black arrows**). There is minimal to mild lymphocyte depletion in the VT68 image (**black asterisk**).

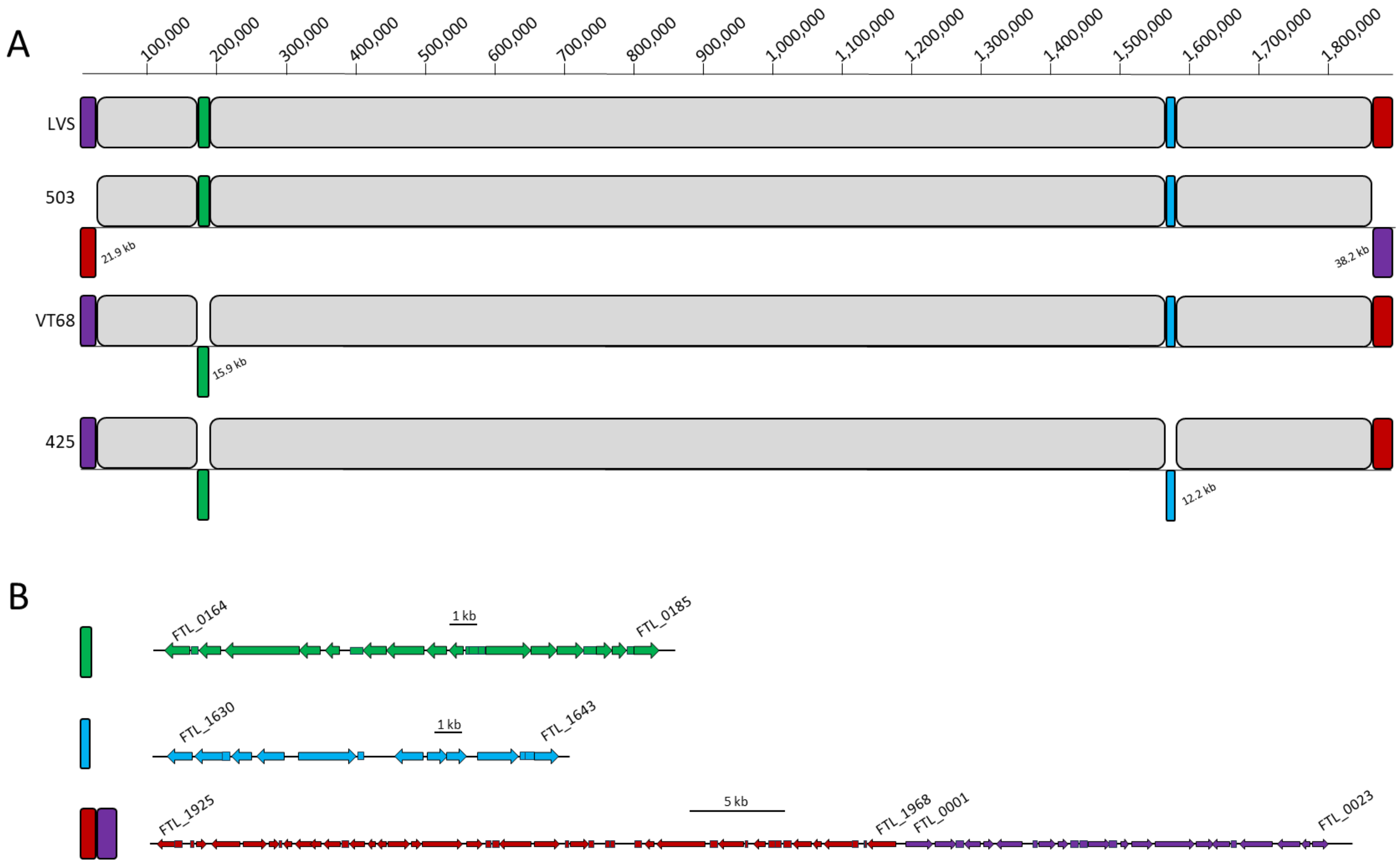

Figure S3. Detection of genomic rearrangements in Type B isolates relative to LVS. A) Mauve was used to generate whole genome alignments compared to LVS. Areas of inversion are shown by color box with the approximate size in kb given. **B)** For comparison purposes, LVS genome co-ordinates are show

Type B test panel: Supplementary Materials

TABLES:

Table S1: Average nucleotide identity of VT68, 425, and 503 using Schu S4 as a reference.

	LVS	VT68	425	503	Schu S4
**LVS**	100.0000				
**VT68**	99.9216	100.0000			
**425**	99.9223	99.9088	100.0000		
**503**	99.9853	99.9198	99.9198	100.0000	
**SchuS4**	99.3444	99.3401	99.3431	99.3407	100.0000

Table S2: SNPs identified in VT68, 425, and 503 relative to the LVS genome.

Associated as an .xlsx file.
